# Molecular Determinants of S100B Oligomer Formation

**DOI:** 10.1371/journal.pone.0014768

**Published:** 2011-03-18

**Authors:** Eva Thulin, Tõnu Kesvatera, Sara Linse

**Affiliations:** 1 Department of Biochemistry, Lund University, Lund, Sweden; 2 Laboratory of Bioorganic Chemistry, National Institute of Chemical Physics and Biophysics, Tallinn, Estonia; Swiss Federal Institute of Technology Lausanne, Switzerland

## Abstract

**Background:**

S100B is a dimeric protein that can form tetramers, hexamers and higher order oligomers. These forms have been suggested to play a role in RAGE activation.

**Methodology/Principal Findings:**

Oligomerization was found to require a low molecular weight trigger/cofactor and could not be detected for highly pure dimer, irrespective of handling. Imidazol was identified as a substance that can serve this role. Oligomerization is dependent on both the imidazol concentration and pH, with optima around 90 mM imidazol and pH 7, respectively. No oligomerization was observed above pH 8, thus the protonated form of imidazol is the active species in promoting assembly of dimers to higher species. However, disulfide bonds are not involved and the process is independent of redox potential. The process was also found to be independent of whether Ca^2+^ is bound to the protein or not. Tetramers that are purified from dimers and imidazol by gel filtration are kinetically stable, but dissociate into dimers upon heating. Dimers do not revert to tetramer and higher oligomer unless imidazol is again added. Both tetramers and hexamers bind the target peptide from p53 with retained stoichiometry of one peptide per S100B monomer, and with high affinity (lgK = 7.3±0.2 and 7.2±0.2, respectively in 10 mM BisTris, 5 mM CaCl_2_, pH 7.0), which is less than one order of magnitude reduced compared to dimer under the same buffer conditions.

**Conclusion/Significance:**

S100B oligomerization requires protonated imidazol as a trigger/cofactor. Oligomers are kinetically stable after imidazol is removed but revert back to dimer if heated. The results underscore the importance of kinetic versus thermodynamic control of S100B protein aggregation.

## Introduction

S100B belongs to the S100 family of small calcium-binding proteins within the calmodulin superfamily of EF-hand proteins. Over 20 different S100 proteins are known and all members of this family contain two helix-loop-helix calcium-binding motifs. Most S100 proteins are known to form a homodimer, or a heterodimer with another S100 member, and interact with a wide range of proteins involved mainly in the cytoskeleton and cell proliferation. Many S100 proteins have strong medical implications in, for example, inflammatory response, rheumatoid arthritis, cancer, and neurological conditions. For extensive reviews, see [1]-[5]. The name S100 refers to the fact that these proteins are soluble in 100% saturated ammonium sulphate solution.

S100B is found in vertebrates only and has regulatory activities both as an intracellular and an extracellular protein. S100B is abundant within the central nervous system, for example in glia [6]. In cells, S100B is involved in signal transduction because it inhibits protein phosphorylation, regulates enzyme activities and is involved in Ca^2+^ homeostasis. S100B interacts with cytoskeletal proteins and is involved in the regulation of cellular morphology. Extracellularly, S100B is present in for example serum and spinal fluid. Here the protein seems to act as a beneficial factor when present at low (nM) concentration and stimulates neurite outgrowth and cell survival through interactions with the receptor of advanced glycation end products, the RAGE receptor [7], [8]. S100B seems to both stimulate myoblast proliferation and inhibit myoblast differentiation [9]. Under certain conditions, e.g. after cardiac surgery, cardiac arrest, stroke, or head injury, S100B leakage into the extracellular fluids increases to toxic levels [10]. It seems like in this situation, the interaction with RAGE underlies the toxic effects of S100B [7]. The concentration of S100B is used in diagnostics to predict the severity of brain damage, e.g. after cardiac surgery [4], [11].

S100B levels are elevated in the brains of patients with Alzheimer's disease [12] or Down's syndrome, and the protein is implicated in cancer. In Alzheimer's disease the pattern of S100B overexpression correlates with the pattern of neuritic-plaque formation. Increased amounts of S100B are also detected in tumours, e.g. in malignant melanoma [13] and the S100B concentration correlates with the severity of the disease. S100B binds to p53 and inhibits the calcium-dependent phosphorylation of p53 by protein kinase C. This may reduce the tumour suppressor function of p53, resulting in uncontrolled tumour growth. S100B has also been found to affect gene expression through interactions with transcription factors [14]. The p53 transcriptional activity is inhibited by S100B, and down-regulation of S100B is a means to restore p53 levels [15], [16].

S100B is a calcium-sensor protein, and upon Ca^2+^ binding it exposes hydrophobic surfaces that interact with target proteins [17]-[20]. S100B binds two Ca^2+^ ions per monomer (10.7 kDa), and Ca^2+^ affinities are altered in the presence of the target [21]. S100B is also a high-affinity Zn^2+^-binding protein [22]. Three-dimensional structures of S100B are reported for the calcium free [23] and calcium bound [24]-[26] states, with both calcium and zinc bound [27], as well as in complex with a range of peptides from target proteins [28]-[30]. All these structures display S100B as a homodimeric protein (21.5 kDa) and the affinity is so high, with K_D_ in the picomolar range or lower [31] that monomers are absent under all relevant biological circumstances. S100B homodimerization occurs mainly via the terminal helices, and the dimer is not 3D-domain swapped, in contrast to the dimer found for S100G (calbindin D_9k_) [32]. S100G is the only S100 protein that is observed as a monomeric protein under physiological conditions.

The high resolution structure of a S100B octamer has recently been reported and tetramers were proposed to trigger RAGE activation by receptor dimerization. [33]. Here we describe the independent discovery of S100B tetramers, hexamers and higher order oligomers. Using highly purified dimers in the absence and presence of potential catalyzing agents, we have determined the conditions dictating their formation and disassembly. We show that conversion from dimer to higher oligomers requires a low molecular weight trigger, and that protonated imidazol can serve as this trigger. In addition we quantify the p53 target binding properties of tetramers and hexamers in comparison with dimer.

## Results

### S100B oligomerization requires a trigger

S100B tetramers and higher order oligomers were discovered by serendipity during our initial purification of recombinant rat S100B from *E coli* using heat treatment, ion exchange and size exclusion chromatography (see File S1). The formation of S100B tetramers and higher oligomers could be due to the very high concentration of S100B and/or lyophilization of the protein during purification. Alternatively, the formation of tetramers could be due to a trigger, which could be an *E coli* protein or some other substance from *E coli*, or any of the buffer chemicals and salts (imidazol, EDTA, NaCl, ammonium acetate) used during purification. To distinguish between these possibilities, the purification protocol was expanded with hydrophobic interaction chromatography on phenyl sepharose column between the ion exchange and gel filtration steps. After the gel filtration S100B was repeatedly denatured by urea and washed and desalted on a size exclusion filter (cutoff 5 kDa) until no contaminating proteins or small molecules could be detected by gel electrophoresis or NMR spectroscopy. This highly purified (trigger-free) S100B dimer was desalted and lyophylized. A fraction of the lyophylized material was dissolved at 20 mg/ml and split in 24 samples that were supplemented with excess Ca^2+^ or EDTA, with NaCl ranging from no added salt to saturated solution, and with pH adjusted to 5, 6, 7, 8 or 9. The samples were lyophilized and dissolved, and then incubated for 24 hours at 4 °C followed by analytical gel filtration on a Superdex 200 column. Only the dimer peak was observed at all these conditions, ruling out lyophilization as the cause of formation of S100B tetramers and higher oligomers. Neither did we observe any higher Mw species than dimer after concentration of the samples on spin concentrators. This batch of highly pure S100B dimer is hereafter referred to as trigger-free S100B.

These results show that the oligomerization of S100B dimer requires a trigger and is not caused by high concentration of the protein alone. Lyophilization or other means of concentration of trigger-free S100B dimer does not yield any other species than dimer.

### Localization of the trigger along the purification protocol

The next step was to isolate and identify the trigger that lead to the formation of S100B tetramers and oligomers. The S100B eluate from the ion exchange column in the purification protocol was divided into six pools that were separately lyophilized. Each pool was dissolved in the smallest possible volume, incubated at 4 °C for 24 hours and analyzed by analytical gel filtration. The largest amount of tetramer and higher oligomer was obtained from pool six, representing the high salt end of the S100B eluate, implying that the trigger was most abundant in this pool. To further isolate the trigger, pool six was purified by hydrophobic interaction chromatography on a phenyl sepharose column (binding and washing in Ca^2+^, elution with EDTA). One hundred fractions were collected from the phenyl sepharose flow through, wash and elution, and analyzed by agarose gel electrophoresis. S100B was present in the EDTA eluate but not in the flow-through or wash fractions. Analytical gel filtration showed that the protein was present as a dimer in the EDTA eluate. Fractions from the EDTA eluate were lyophilized, dissolved, incubated for 24 h and analysed by analytical gel filtration, but no tetramer or higher oligomer was obtained from any of these fractions. Therefore, trigger-free S100B dimer was mixed with aliquots from flow-through and wash fractions, lyophilized, dissolved, incubated for 24 h and analysed by gel filtration. Tetramers and higher oligomers were obtained when trigger-free S100B was mixed with the flow through fractions, but not the wash fractions, and there was a clear maximum of potency to trigger S100B oligomerization in fraction 33 representing the late flow through of the phenyl sepharose column.

The trigger was isolated in fraction 33 and remained to be identified.

### High or low Mw trigger?

The trigger could be another protein, DNA or biological macromolecule present in *E. coli*, or a low Mw substance from *E. coli* or an added chemical or buffers. Phenyl sepharose fraction 33 was therefore fractionated using a 5 kDa Mw cutoff filter. The concentrate (high molecular weight species, Mw>5 kDa) and filtrate (low molecular weight substances, Mw<5 kDa) were lyophilized separately with trigger-free S100B dimer, dissolved, incubated for 24 h and analysed by analytical gel filtration as above. S100B oligomers were obtained when the protein was incubated with the filtrate, but not the concentrate, implying that the trigger is a low M_w_ substance (<5 kDa).

We found that the trigger is a low Mw substance.

### Identification of the trigger

**Figure 1:**
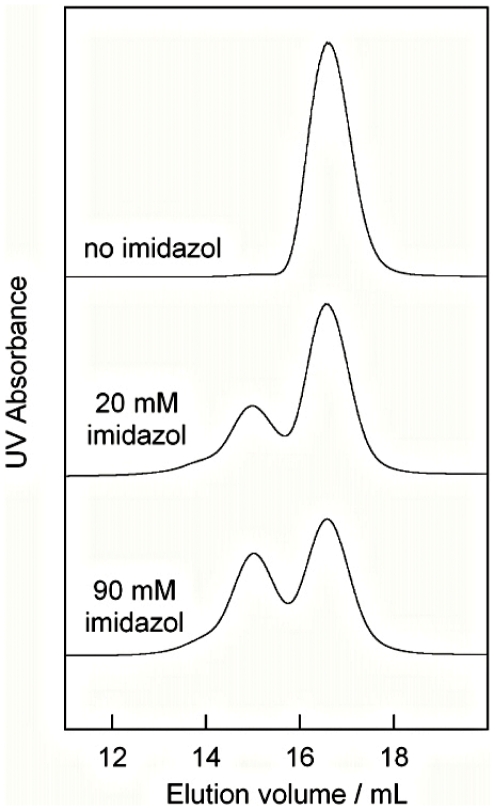
Identification of the trigger. Highly pure S100B dimer was mixed with potential triggers and lyophilized, dissolved, incubated for 24 h at 4 °C, and analyzed by analytical gel filtration on a Superdex 200 column at room temperature. The chromatograms shown are for samples with 20 mg/ml S100B, 1 mM EDTA, 1 mM DTT, 0.25 M NaCl, pH 7.0, and no (top), 20 mM (middle) or 90 mM imidazol .

The concentrate and filtrate from phenyl sepharose fraction 33, as well as aliquots of all fractions from the ion exchange and phenyl sepharose columns, were lyophylized and analyzed by ^1^H NMR in D_2_O and in CDCl_3_. A large number of substances were seen in all fractions. The only NMR signals obtained in D_2_O, which correlated with the oligomerization propensity were the characteristic intense signals from EDTA and imidazol. The only NMR signals obtained in CDCl_3_, which correlated with the oligomerization propensity were a sharp singlet at 1.26 ppm and a much smaller signal at 0.88 ppm, features typical for very long chain alkanes, alcohols and fatty acids (SBDS http://www.aist.go.jp/RIODB/SDBS/menu-e.html). EDTA, imidazol and a number of alkanes, alcohols and fatty acids with different chain lengths were therefore evaluated in triggering experiments where they were added alone and in different combinations to trigger-free S100B dimer at pH 7.0 with or without NaCl up to saturating concentration. Imidazol was identified as the substance that promotes formation of tetramers (Figure 1). No samples devoid of imidazol yielded any oligomeric species higher than dimer.

Imidazol was identified as the trigger substance that promotes formation of S100B tetramer and higher oligomers.

### Tetramers contain no bound trigger

**Figure 2:**
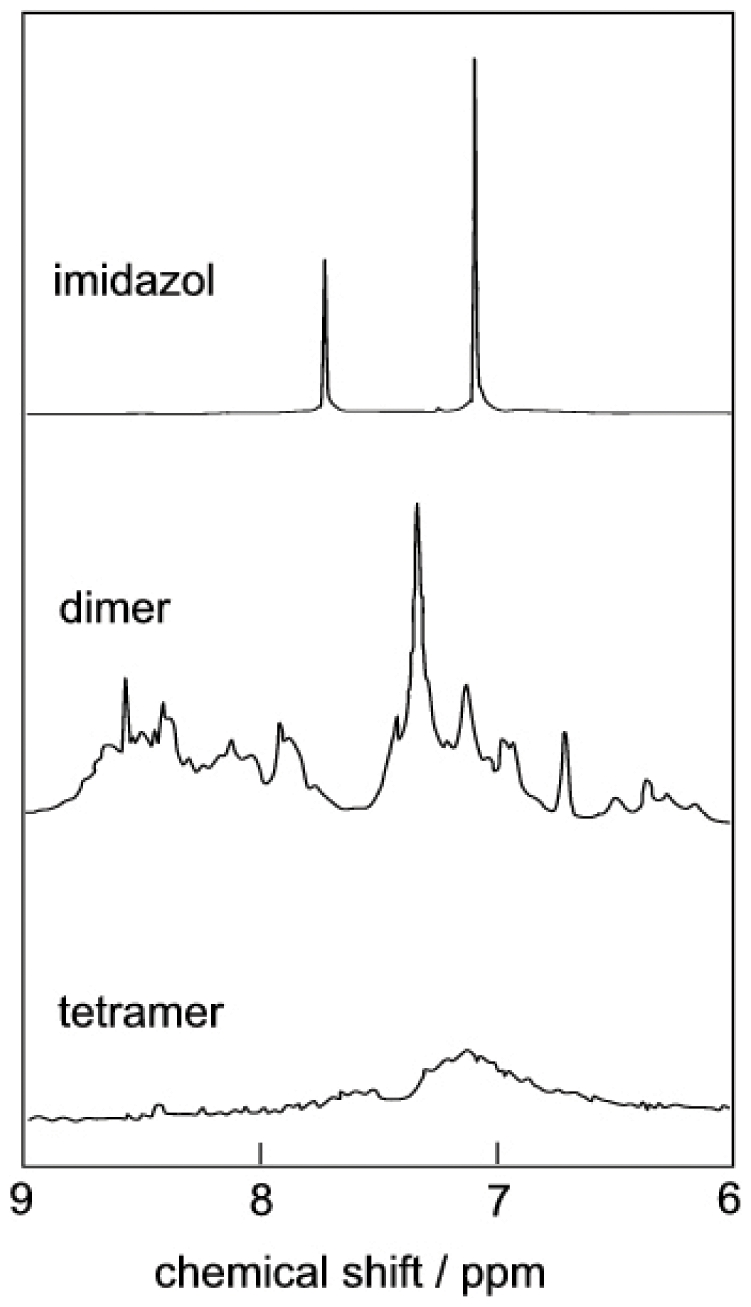
NMR spectra reveal no imidazol in S100B tetramer or dimer isolated by gel filtration. ^1^H NMR spectra in D_2_O for imidazol and the dimer and tetramer fractions as purified by gel filtration after triggering of 20 mg/ml S100B with 90 mM imidazol in1 mM EDTA, 1 mM DTT and 0.25 M NaCl.

We next asked whether imidazol acts as a catalyst or whether tetramers and oligomers are stabilized by bound imidazol. Trigger-free S100B dimer was used in triggering experiments with imidazol (20 mg/ml S100B, 90 mM imidazol, 1 mM EDTA, 1 mM DTT, pH 7.0). The higher oligomer, hexamer, tetramer and dimer fractions were collected separately from the superdex 200 column, lyophilized, dissolved in D_2_O and subjected to NMR spectroscopy in comparison with imidazol. As shown in Figure 2, no imidazol signals were observed in the dimer or tetramer fractions collected from the gel filtration column. Neither was there any imidazol in the collected hexamers or higher oligomer fractions (data not shown). Our data show that the tetramers and higher oligomers are free from imidazol, hence they are not stabilized by bound imidazol.

We found that imidazol acts as a catalyst that promotes the formation of tetramer and higher oligomer, but once formed these do not require imidazol to persist.

### Role of pH and imidazol concentration

**Figure 3:**
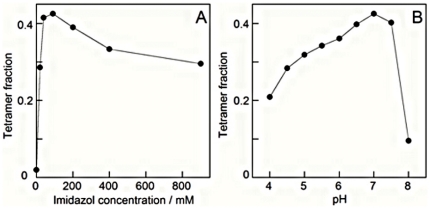
Tetramer formation - dependence on imidazol concentration and pH. Analytical gel filtration on a Superdex 200 column was used to estimate the fraction of S100B in tetrameric state after triggering experiments starting with 20 mg/ml highly pure S100B dimer in 1 mM EDTA, 1 mM DTT and 0.25 M NaCl with different concentrations of imidazol at pH 7.0 (A) or with 90 mM imidazol at different pH values (B).

The role of imidazol concentration was investigated in triggering experiments with 20 mg/ml trigger-free S100B dimer, 1 mM EDTA, 1 mM DTT, 0.25 M NaCl, pH 7 and none or 20–900 mM imidazol. Figure 1 shows examples of chromatograms for zero, 20 and 90 mM imidazol at pH 7. Clearly, the amount of tetramer formed is dependent on the imidazol concentration, as presented in more detail in Figure 3A. The role of pH investigated in triggering experiments with 20 mg/ml trigger-free S100B dimer in 90 mM imidazol, 1 mM EDTA, 1 mM DTT, 0.25 M NaCl at different pH values ranging from 4 to 9. There is a clear dependence on pH (Figure 3B), with an optimum found at pH 7 which coincides with the pKa value of imidazol. Tetramerization is eliminated at pH values above 8 where imidazol is unprotonated and has no charge. Replacement of imidazol with Tris buffer did not yield any tetramers or higher oligomers (not shown).

The amount of tetramer formed is dependent on the imidazol concentration with an optimum around 90 mM and on the pH of the solution with an optimum around pH 7.

### Role of NaCl, redox potential, Ca^2+^ and Zn^2+^

The role of NaCl concentrations was investigated in triggering experiments with 20 mg/ml trigger-free S100B dimer, 1 mM EDTA, 1 mM DTT, 90 mM imidazol, pH 7 and none or 10 mM – saturating NaCl. No effect of the NaCl concentration was seen (data not shown). The role of Ca^2+^ concentrations was investigated in triggering experiments with 20 mg/ml trigger-free S100B dimer, 90 mM imidazol, 1 mM DTT, 0.25 M NaCl, pH 7 and 1–10 mM CaCl_2_ or 1–10 mM EDTA. No effect of the Ca^2+^ concentration was seen (data not shown). The effecy of redox potential was investigated in triggering experiments with 20 mg/ml trigger-free S100B dimer, 1 mM EDTA, 90 mM imidazol, pH 7 and either 1 or 10 mM DTT or 10 mM gluthathione at different ratios of reduced and oxidized species. No effect of the redox potential was seen (data not shown), hence disulfide bond formation is not involved in the oligomerization process. Imidazol is a zinc chelator, and the effect of zinc was therefore investigated by preparing samples of trigger-free S100B dimer at pH 7.0 with 1 mM DTT and 0.25 M NaCl, and different combinations of Zn^2+^ and imidazol. Each sample was lyophylized, dissolved, incubated for 24 h and analysed by analytical gel filtration. Zn^2+^ was found to casue no extra effect compared to the same imidazol concentration without Zn^2+^.

None of the factors examined governed the amount of tetramer or higher oligomer formed.

### Comparison of imidazol to related substances

The role of imidazol in comparison to the related substances histidine, methylimidazol and poly-histidine was investigated in triggering experiments with trigger-free S100B dimer in 1 mM EDTA, 1 mM DTT, 0.25 M NaCl, pH 7.0 with (0–100 mM) imidazol, 50, 100, 200, 400, or 800 mM histidine, 50, 100, 200, 400, or 800 mM methylimidazol or 0.1, 0.2, 0.4, 0.8 or 1.6 mM poly-histidine of mean chain length 100 residues. Imidazol was the only substance found to cause the formation of tetramer and higher oligomer, while only dimer was observed when S100B was incubated with histidine, methylimidazol or poly-histidine (data not shown).

The triggering effect of imidazol was not seen with related substances.

### S100B tetramers are kinetically stable

**Figure 4:**
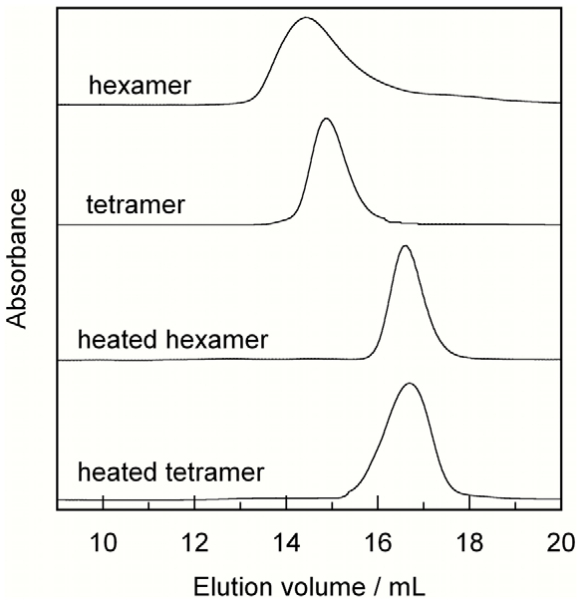
Kinetic stability. An imidazol-triggered sample of S100B was separated into hexamer, tetramer and dimer by gel filtration and the fractions reinjected on the Superdex 200 analytical gel filtration column before (top two chromatograms) and after (bottom two chromatograms) heating to 90 °C for 15 minutes.

Trigger-free S100B dimer was used in triggering experiments with imidazol (20 mg/ml S100B, 90 mM imidazol, 1 mM EDTA, 1 mM DTT, pH 7.0). The hexamer, tetramer and dimer fractions were collected separately from the superdex 200 column and an aliquot of each fraction reinjected on the column (Figure 4). The fractions stored for up to 6 months during which time aliquots were repeatedly analyzed by analytical gel filtration on the superdex 200 column. The tetramers were found persist for six months in solution at 4 °C (longer times not tested). The hexamer showed faster conversion back to dimer with approximately 50% lost after three months of storage at 4 °C. The dimer remained dimeric for the entire period. The time-dependent behavior was repeated twice using different batches of oligomer, and the same result was found. Aliquots of the newly collected tetramer and hexamer fractions were also heated for 15 minutes at 90 °C prior to analytical gel filtration. The heating procedure was found to dissociate tetramers and hexamers into dimers (Figure 4). Aliquots of the heated samples were then incubated at 4 °C for up to 7 days and again subjected to gel filtration; however neither tetramers nor higher oligomers were regained (not shown). The heating experiments were repeated five times on different batches and dissociation into dimer was seen in all cases. We also found that the tetramers and hexamers as isolated by gel filtration cannot be lyophilized to increase their concentration because this procedure causes a significant amount of the protein to revert to dimer. However, the collected multimers can be concentrated in spin concentrators without causing any dissociation into dimers (data not shown).

We found that the S100B tetramers are kinetically stable over several months, but revert to dimer after thermal denaturation.

### Dynamic light scattering

**Figure 5:**
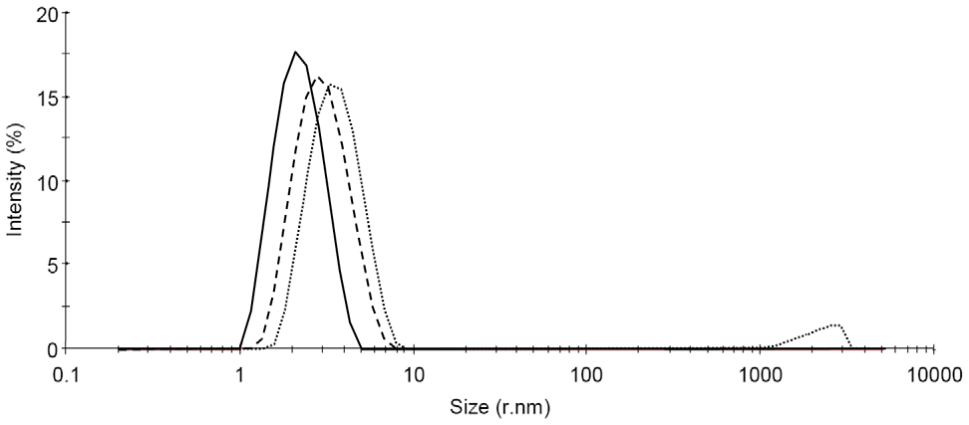
Dynamic light scattering. An imidazol-triggered sample of S100B was separated into hexamer (dotted line), tetramer (dashed line) and dimer (solid line) by gel filtration and the fractions analyzed by dynamic light scattering.

Trigger-free S100B dimer was used in triggering experiments with imidazol (20 mg/ml S100B, 90 mM imidazol, 1 mM EDTA, 1 mM DTT, pH 7.0). The hexamer, tetramer and dimer fractions were collected separately from the superdex 200 column and an aliquot of each fraction subjected to size determination by dynamic light scattering (Figure 5). For the dimer fraction we observe one peak with mean radius of 2.28 nm, which for a globular protein corresponds to a Mw of 22 kDa (21.49 expected for S100B dimer). For the tetramer fraction we observe one peak with mean radius of 3.10 nm, which for a globular protein would correspond to a Mw of 47.5 kDa (43.0 expected for S100B tetramer). The slightly higher Mw compared to the one expected may be due to some contamination with hexamer or may reflect that the shape is not perfectly globular. For the hexamer fraction we observe two peaks. The major peak (94% of the intensity) occurs at mean radius 3.66 nm, which for a globular protein would correspond to a Mw of 70.1 kDa (64.5 expected for S100B hexamer). Again, the slightly higher Mw compared to the one expected may be due to some contamination with higher oligomeric species or that the shape is not perfectly globular. The second peak (6 % of the intensity) occurs at very large radius and may represent further aggregation of a small fraction of the protein.

### S100B oligomers bind p53 target peptide with high affinity

**Figure 6:**
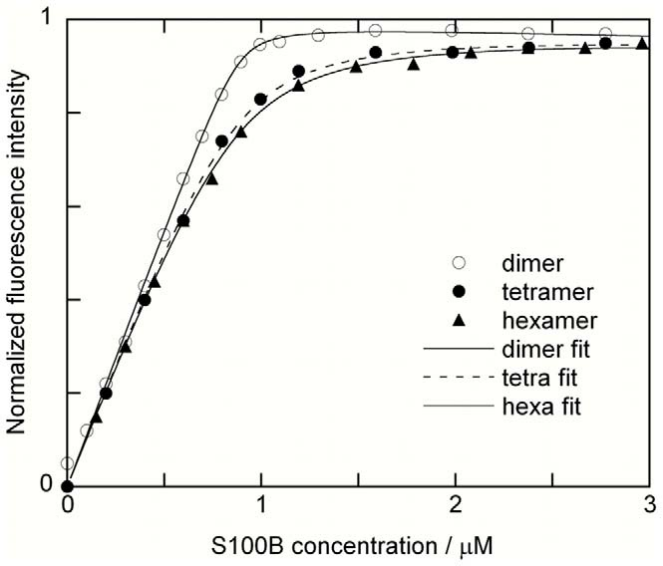
p53 binding. Titration of 0.9 µM p53 peptide with S100B dimer (O), tetramer (•), or hexamer (▴), together with fitted lines. The fluorescence intensity recorded during each titration has been normalized.

We next asked if the S100B oligomers retain their binding affinity and stoichiometry for target peptides that are known to interact with high affinity with the S100B dimer. Fluorescence spectroscopy was used to measure the affinity and stoichiometry of binding between S100B oligomers and the target peptide from p53. The peptide used contains residues 367–388 of p53 with phenylalanine 385 changed to a tryptophan to enable the fluorescence studies [19]. The fluorescence emission intensity at 330 nm was monitored during stepwise addition of S100B dimer, tertramer or hexamer stock solution. The fluorescence intensity was plotted *versus* S100B concentration and a 1:1 binding model was fitted to the data, as illustrated in Figure 6. The data show that all three forms of S100B bind one p53 peptide per S100B monomer, i.e. two peptides per dimer, four peptides per tetramer and six peptides per hexamer. The peptide-binding affinity is similar in all three cases, with ^10^log K values of 8.2±0.3 for dimer, 7.3±0.2 for tetramer, and 7.2±0.2 for hexamer.

We find that p53 binding is largely unaffected (one order of magnitude only) by the association of S100B dimers into tetramers and higher oligomers.

## Discussion

S100B has been known for long time to form a high affinity homodimer. Here we have reported the discovery of S100B tetramers and higher oligomers, which were found independently by Ostendorp et al. [33]. Using a combination of classical protein chemistry and NMR spectroscopy and highly pure S100B dimer we show that the conversion of S100B dimer into tetramer and higher oligomers requires a low Mw trigger. Imidazol was identified as a molecule that can serve as the trigger, with a pH optimum at pH 7 which coincides with the pKa value of imidazol.

The pH dependence data clearly show that imidazol is effective as a trigger in its protonated (positively charged) state, because the activity is lost at pH values above 8 where imidazol in uncharged. The more modest loss of triggering activity observed as pH is lowered between 7 and 4.5 is most likely due to protonation of ionizable groups in the S100B protein. The failure of other buffers with pKa values in the same range as imidazol to trigger the oligomerization suggests a specific role for imidazol. When isolated by gel filtration, both tetramers and higher oligomers are found to be free of imidazol, hence they are not stabilized by binding the trigger. The absence of bound imidazol in the purified tetramers and hexamers shows that imidazol facilitates the assembly of tetramers and higher oligomers; however imidazol is not essential for maintaining these structures once formed. The oligomers are kinetically stable towards dissociation into dimers, with tetramers being the most stable form. However, if the purified tetramers or hexamers are denatured by heat and then cooled, they revert to dimer, which agrees with the observation by NMR spectroscopy that the triggering imidazol is not present in these species.

Imidazol is commonly used in protein purification. For example when poly-His-tags are used for purification purposes it is common to use high concentration of imidazol in protein elution steps. The present findings suggest that the His-tag may not be the only cause of unexpected effects in such protein preparations, but also imidazol may contribute. As shown in the present work, the protein alterations caused by imidazol may persist after imidazol is removed. For example, it was recently found that imidazol significantly accelerates the rate of ground state recovery of the flavin chromophore the BLUF domain of the protein AppA [34], but in contrast to S100B tetramerization, the unprotonated form of imidazol was found to be the active species in that case.

The higher affinity binding to RAGE of S100B tetramers compared to S100B dimers, was inferred to couple to RAGE dimerisation, hence allosteric couplings may explain the higher affinity in that case [33]. All substances tested here besides imidazol fail to induce the formation of tetramers or higher oligomers. This includes even the related substances histidine, methyl-imidazol and polyhistidine. Thus it remains to be found what causes the formation of tetramers *in vivo*, if there is another low molecular weight trigger or whether interaction with targets like for example the RAGE receptor is involved. The higher affinity of RAGE for S100B tetramer compared to dimer, implies through thermodynamic linkage that the dimer to tetramer equilibrium constant is higher in the presence of RAGE. Here we find that binding of the target peptide from the tumour suppressor protein p53 is largely retained in tetramers and hexamers, both as regards the affinity and stoichiometry. The effect on affinity is less than an order of magnitude and there is one p53 peptide bound per S100B monomer both in dimer, hexamer and tetramer. This implies that the tetramers and hexamers are functional and that the target-binding sites are largely unperturbed in these structures.

## Materials and Methods

### Protein Expression

The plasmid with the rat S100B gene (kind gift from D. Weber) was transformed in *Eschericia coli* BL21 De3 PLysS star and grown in LB medium with 50 mg/l ampicillin and 30 mg/l chloramphenicol. 5 ml of an overnight culture was used to inoculate a 500 ml day culture in a 2.5 l baffled flask in a shaker incubator at 37 °C. IPTG was added at OD600 = 0.6, and the cells were collected 3–4 hours later by centrifugation at 6000 g for 5 minutes, and frozen.

### Purification of highly pure S100B dimer

The cell pellet is sonicated on ice in buffer A (10 mM imidazol, 1 mM EDTA, pH 7.0; 40 ml buffer to pellet from 500 ml culture) and centrifuged at 27000 g for 10 minutes. The supernatant is poured into an equal volume of boiling buffer A, heated to 80 °C and cooled on ice. Precipitated *E. coli* proteins are removed by centrifugation at 15000 g for 10 minutes, and the supernatant is pumped onto a DEAE cellulose column in buffer A, washed with buffer A and eluted with a linear salt gradient from 0 to 300 mM NaCl in buffer A. The eluted fractions are analysed by agarose gel electrophoresis and the S100B-containing fractions are pooled, supplemented with 2 mM CaCl_2_ and 300 mM NaCl and pumped onto a phenyl sepharose column packed in buffer B (10 mM Tris, 1 mM CaCl_2_, 300 mM NaCl, pH 7.5). The column is washed with buffer B and S100B dimer is eluted with 10 mM Tris, 1 mM EDTA, 300 mM NaCl, pH 7.5. The eluate is freeze dried and dissolved in the smallest possible volume before gel filtration on a 3.4×200 cm Sephadex G50 superfine column with 50 mM ammonium acetate, pH 6.5 as the running buffer. Fractions containing dimeric S100B are pooled and lyophilized. The protein is then dissolved in 8M urea with 10 mM EDTA, pH 8.0, and incubated for 2 hours to release any small molecule inclusions. The protein is washed on a spin concentrator (Mw cutoff 5 kDa), first with 8M urea, then with H_2_O, and lyophylized. This denaturation and washing procedure is repeated until no contaminating species could be detected by gel electrophoresis or NMR spectroscopy and no tetramer or higher oligomer is obtained after lyophilization. The sample is then considered free from triggering molecules from *E. coli* and can be used in oligomerization experiments under controlled conditions.

### P53 peptide expression and purification

A peptide based on the C-terminal regulatory domain of p53 with the sequence MSHLKSKKGQSTSRHKKLMWKTE (Met followed by residues 367–388 of p53, with Phe 385 changed to Trp), was cloned in the vector pTXb1 from New England Biolabs. Overlapping oligonucleotides representing the forward and reverse strands with the required overhangs were heated to 95 °C and annealed by slow cooling (2 °C per minute) to 60 °C, then rapidly cooled to 4 °C. The product was ligated with pTXb1 vector that had been cut with Nde1 and SapI restriction enzymes, transformed in *E. coli* ER2566, and spread on LB plates with 100 µg ampicillin per ml. DNA sequencing was performed on plasmids prepared from single colonies. A plasmid with confirmed sequence was then transformed into *E. coli* ER2566 and the peptide was expressed in fusion peptide with an intein and a chitin-binding domain. The fusion product was purified by sonication, ion exchange on a DEAE cellulose resin in 10 mM Tris/HCl, 1 mM EDTA, pH 7.5, using step elution with the same buffer complemented with 0.5 M NaCl, then binding to and washing on a chitin column in 10 mM Tris/HCl, 0.5 M NaCl, 1 mM EDTA, pH 7.5. The peptide was cleaved off from the intein using the same buffer supplemented with 50 mM DTT. The peptide was then further purified by gel filtration on a G25 column, desalted and lyophilized.

### Chemicals

All buffers and purification chemicals were of analytical grade. The following potential triggers were purchased from Sigma-Adrich: alkanes ranging from tetradecane (C_14_H_30_) to octatriacontane (C_38_H_78_), alcohols ranging from tetradecanol (C_14_H_30_O) to octacosanol (C_28_H_58_O), fatty acids from tetradecanoic acid (C_14_H_28_O_2_) to octacosanoic acid (C_28_H_56_O_2_).

### Triggering experiments

Highly pure S100B dimer was used in all triggering experiments. The protein solution was supplemented with a range of different pure substances (NaCl, EDTA, imidazol, Tris, histidine, methylimidazol, poly-L-histidine, alkanes ranging from tetradecane (C_14_H_30_) to octatriacontane (C_38_H_78_), alcohols ranging from tetradecanol (C_14_H_30_O) to octacosanol (C_28_H_58_O), fatty acids ranging from tetradecanoic acid (C_14_H_28_O_2_) to octacosanoic acid (C_28_H_56_O_2_)), or with lyophilized phenyl sepharose fractions, and set to pH 7. In a separate series of experiments, highly pure S100B dimer was supplemented with 90 mM imidazol at pH values ranging from 4–9. Each sample was lyophilized and then dissolved in H_2_O to obtain 20 mg/ml S100B and incubated for 24 h at 4 °C and analysed by analytical gel filtration on a Superdex 200 column.

### Analytical gel filtration

Analytical gel filtration chromatography was performed using a Superdex200 column (Amersham Biosciences, Uppsala, Sweden) connected to a BioLogic HR system (Biorad). The running buffer was 25 mM sodium carbonate with 25 mM HCl, pH 7.3. The column was calibrated with the following standards: ferritin (440 kDa), Bovine serum albumin (67 kDa), ovalbumin (43 kDa), calbindin D28k (30 kDa), carbonic anhydrase (30 kDa), a-lactalbumin (14 kDa), calbindin D9k (8.5 kDa), the N-terminal domain of calmodulin (8 kDa), and protein G B1 domain (6 kDa). The elution time vs. log(Mw) was reasonably well fitted by a straight line, R = 0.93 (see supplementary information).

### NMR spectroscopy

NMR spectra were obtained at 27 °C at 500 or 600 MHz on a GE Omega 500 (rebuilt to a Varian/Bruker system) or Varian Unity Plus 600 MHz spectrometer. Samples were dissolved in D_2_O or CDCl_3_ and spectra recorded with solvent presaturation. Between 64 and 20000 transients were accumulated depending on the sample concentration.

### Dynamic light scattering

Dynamic light scattering was performed using a ZetaSizer Nano-S instrument from Malvern Instruments (Malvern Worcs, UK). Fractions were collected directly from the Superdex200 gel filtration column in 25 mM sodium carbonate with 25 mM HCl, pH 7.3, and studied in single use plastic cuvettes to avoid contamination by dust or other impurities.

### Titration of p53 peptide with S100B

Binding parameters were obtained from titrations of p53 peptide with S100B dimer, tetramer or hexamer in 10 mM BisTris buffer with 5 mM CaCl_2_ at pH 7.The peptide concentration was between 0.07 and 1.3 µM, and S100B aliquots were added from a concentrated stock solutions. Fluorescence emission at 330 nm (with excitation at 295 nm) was recorded on a Perkin Elmer Luminescence Spectrometer LS50B connected to a Julabo F25 thermostatic water bath set at 25 °C. Each titration point was obtained by integration of the signal over 30 s after 1–1.5 minutes of equilibration. The peptide concentration was determined using absorbance spectroscopy and the S100B monomer concentration in each stock solution was determined using amino acid analysis after acid hydrolysis (analysis purchased from BMC, Uppsala). A 1:1 binding model was fitted to the fluorescence as a function of total S100B monomer concentration using CaLigator software [35]. Data at 0.07–0.9 µM p53 peptide were used to determine the K_D_. The affinity as well as protein stock concentration were used as variable parameters in the fits. In all cases (dimer, tetramer and hexamer), there was a very good agreement between the fitted protein concentration and that obtained from acid hydrolysis, meaning that all species bind one p53 peptide per S100B monomer. The reported binding constants are averages of at least three independent measurements.

## Supporting Information

File S1 Supplementary Text and Figures
(0.18 MB PDF)
