# Supplementary material for: Molecular Determinants of S100B Oligomer Formation
Source: PLoS One. 2011 Mar 18;6(3):e14768. doi: 10.1371/journal.pone.0014768 (PMC3060798; doi:10.1371/journal.pone.0014768)
Supplement: File S1 — Supplementary Text and Figures (0.18 MB PDF) [file pone.0014768.s001.pdf]

Molecular determinants of S100B oligomer formation

Eva Thulin, Tõnu Kesvatera, Sara Linse

Supplementary material

Discovery of S100B tetramer and higher oligomers

During our first purification of recombinant rat S100B expressed in *E. coli*, we found that the protein also forms tetramers and higher order oligomers. This is in part due to the very high yields of protein expressed in our bacterial cultures. We used a purification protocol similar to the second one in the report by van Eldik et al [1], but used sonication instead of lysozyme treatment to break the cells and imidazol instead of Tris buffer in the initial steps. After sonication, the protein was purified by heat treatment and anion exchange chromatography. Cell pellet was sonicated on ice in buffer A (10 mM imidazol, 1 mM EDTA, pH 7.0; 40 ml buffer to pellet from 500 ml culture) and centrifuged at 15000 g for 10 minutes. The supernatant was poured into an equal volume of boiling buffer A, heated to 80 ^{o}C and cooled on ice. Precipitated *E. coli* proteins were removed by centrifugation at 27000 g for 10 minutes, and the supernatant was pumped onto a DEAE cellulose column in buffer A, washed with buffer A and eluted with a linear salt gradient from 0 to 300 mM NaCl in buffer A. The eluate was lyophilized. S100B was found to exhibit an unusually broad elution in the linear salt gradient from the DEAE cellulose column. Therefore, the pooled and lyophilized S100B-containing fractions could not be dissolved in the maximum volume (25 ml) of deionised water that can be applied to our gel filtration column (3.4 cm x 200 cm Sephadex G50 superfine) to obtain a reasonable separation. Instead, the material was dissolved in 50 ml water (yielding a protein concentration of ca. 20 mg/ml), and half of this volume was applied to the gel filtration column (packed in and eluted with 50 mM ammonium acetate buffer at pH 6.5). The second sample was applied to the column 24 hours after the first one.

To our surprise the two chromatograms (Figure S1A) were not identical. In addition to the major peak at the expected dimer position (arrow in Figure S1A), a significant fraction of the protein eluted earlier than dimer consistent with higher molecular weight species. In the first chromatogram, a shoulder appears before the main peak, whereas in the second chromatogram there is an early peak that is clearly separated from dimer. SDS PAGE and agarose gels reveal that both the higher Mw shoulder/peak and the dimer peak contain mainly S100B (Figure S1B and C).

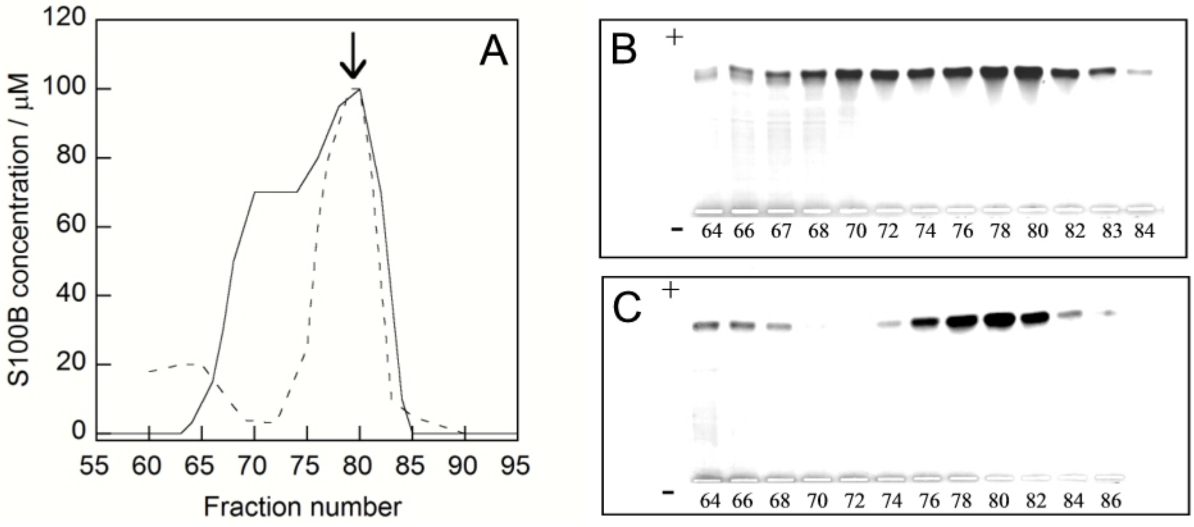

Figure S1: Discovery of S100B multimers. The S100B eluate from an anion exchange DEAE cellulose column was freeze-dried and dissolved in 50 ml H_2O. Half of this solution was applied immediately to a 3.4 x 200 cm gel filtration column (Sephadex G50 superfine) that was run at 4 ^{o}C in 50 mM ammonium acetate buffer pH 6.5, and the S100B content in each fraction was quantified using agarose gel electrophoresis (solid line). The procedure was repeated 24 hours later for the second half of the solution (dashed line). **A.** Chromatograms. The arrow indicates the fraction where dimers are expected to elute from the column based on gel filtration of a large number of different proteins in the Mw range from 6 to 70 kDa on the same column. **B, C**. Agarose gel electrophoresis of selected fractions from the first (B) and second (C) chromatogram.

To obtain better separation, the oligomers as obtained above (fractions 60-74) were pooled and concentrated and 5 ml of the solution was applied to the 3.4 cm x 200 cm (G50 superfine) gel filtration column. Two major oligomer peaks were seen (Figure S2A). Fractions from these two peaks were analysed by analytical gel filtration (Figure 2B). The column was calibrated with proteins with molecular weights ranging from 6 to 440 kDa (Figure S3). Although, there is considerable variation among the standards , the results suggest that the major species in fraction 68 is a hexamer (64.2 kDa), although there is also some tetramer and dimer. Fraction 73 is found to contain mainly tetramer (42.8 kDa), and fraction 80 only dimer (21.4 kDa).

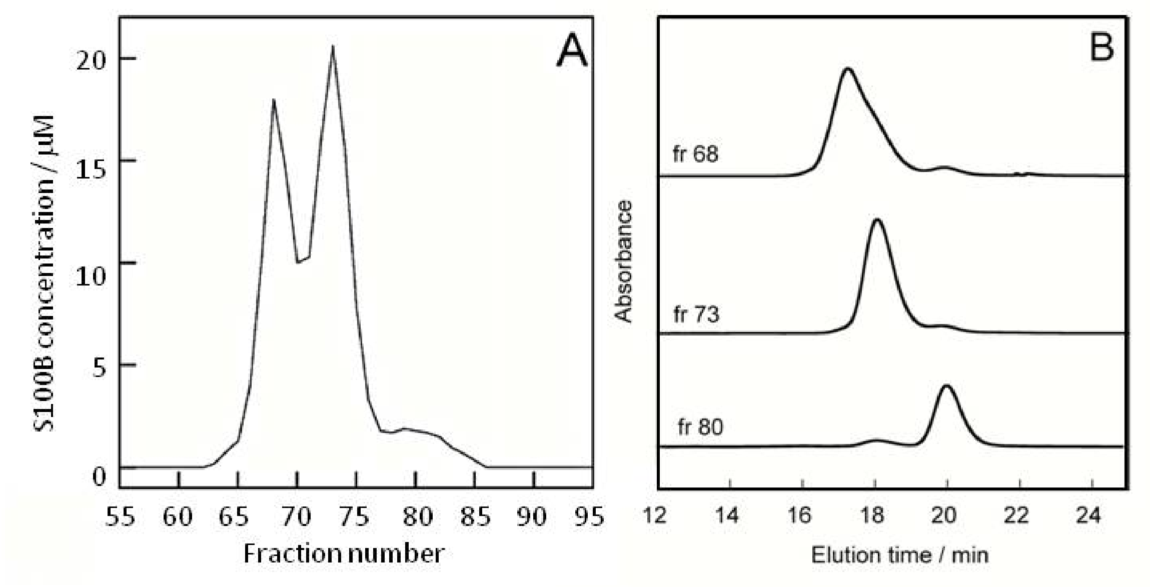

Figure S2. Separation of heaxamers and tetramers from dimers. A. Reinjection of S100B oligomers isolated on a 3.4 x 200 cm gel filtration column (Sephadex G50 superfine at 4 ^{o}C in 50 mM ammonium acetate buffer pH 6.5) on the same column with the S100B content in each fraction quantified using agarose gel electrophoresis. **B.** Analytical gel filtration on a Superdex 200 column of fractions 68, 73 and 80 from the G50 column in panel A. Running buffer 25 mM sodium carbonate with 25 mM HCl, pH 7.3.

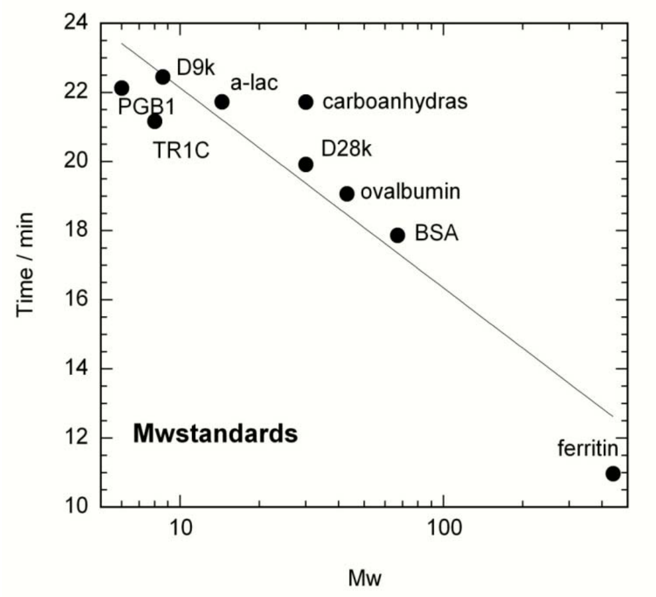

Figure S3. Analytical gel filtration chromatography on the Superdex200 column (running buffer was 25 mM sodium carbonate with 25 mM HCl, pH 7.3) was calibrated with ferritin (440 kDa), Bovine serum albumin (67 kDa), ovalbumin (43 kDa), calbindin D28k (30 kDa), carbonic anhydrase (30 kDa), a-lactalbumin (14 kDa), calbindin D9k (8.5 kDa), the N-terminal domain of calmodulin (8 kDa), and protein G B1 domain (6 kDa). The elution time vs. log(Mw) is fitted by a straight line, R=0.93.

Far-UV CD spectroscopy.

Far-UV CD spectra were recorded between 195 and 250 nm on a JASCO J-720 polarimeter equipped with a Peltier cell thermostated at 25 ^{o}C for hexamer, tetramer and dimer isolated on the superdex 200 column. The spectra are indistinguishable within error limits and shows that all forms of the protein are folded (not shown).

References

1. Van Eldik LJ, Staecker JL, Winningham-Major F. Synthesis and expression of a gene coding for the calcium-modulated protein S100 beta and designed for cassette-based, site-directed mutagenesis. J Biol Chem. 1988 Jun 5;263(16):7830-7.
